# Gain, Loss and Divergence in Primate Zinc-Finger Genes: A Rich Resource for Evolution of Gene Regulatory Differences between Species

**DOI:** 10.1371/journal.pone.0021553

**Published:** 2011-06-29

**Authors:** Katja Nowick, Christopher Fields, Tim Gernat, Derek Caetano-Anolles, Nadezda Kholina, Lisa Stubbs

**Affiliations:** 1 Institute for Genomic Biology, University of Illinois, Urbana, Illinois, United States of America; 2 Department of Cell and Developmental Biology, University of Illinois, Urbana, Illinois, United States of America; Louisiana State University, United States of America

## Abstract

The molecular changes underlying major phenotypic differences between humans and other primates are not well understood, but alterations in gene regulation are likely to play a major role. Here we performed a thorough evolutionary analysis of the largest family of primate transcription factors, the *Krüppel*-type zinc finger (KZNF) gene family. We identified and curated gene and pseudogene models for KZNFs in three primate species, chimpanzee, orangutan and rhesus macaque, to allow for a comparison with the curated set of human KZNFs. We show that the recent evolutionary history of primate KZNFs has been complex, including many lineage-specific duplications and deletions. We found 213 species-specific KZNFs, among them 7 human-specific and 23 chimpanzee-specific genes. Two human-specific genes were validated experimentally. Ten genes have been lost in humans and 13 in chimpanzees, either through deletion or pseudogenization. We also identified 30 KZNF orthologs with human-specific and 42 with chimpanzee-specific sequence changes that are predicted to affect DNA binding properties of the proteins. Eleven of these genes show signatures of accelerated evolution, suggesting positive selection between humans and chimpanzees. During primate evolution the most extensive re-shaping of the KZNF repertoire, including most gene additions, pseudogenizations, and structural changes occurred within the subfamily homininae. Using zinc finger (ZNF) binding predictions, we suggest potential impact these changes have had on human gene regulatory networks. The large species differences in this family of TFs stands in stark contrast to the overall high conservation of primate genomes and potentially represents a potent driver of primate evolution.

## Introduction

Transcription factors (TFs) play a crucial role in regulating the activity of genes and determining phenotypes. Molecular changes in some TFs, for example *FOXP2*, *Runt*, and *PRDM9,* have been linked to the evolution of species differences [1], [2], [3], [4]. Despite their important role, little is known about the TFs in most vertebrate species. In fact, the content of TFs in the human genome has only recently been determined [5] and TFs in other primate genomes remain largely uncharacterized.

In mammalian genomes the largest family of TFs are the zinc finger (ZNF) genes, the majority of which are members of the *Krüppel*-type (C2H2, or KZNF) subfamily. Most of mammalian KZNF genes encode proteins in which one or more N-terminal *Krüppel*-associated Box (KRAB) effector domains are tethered to an array of multiple ZNF motifs [6]. Through interactions with a co-repressor, called KAP-1, KRAB motifs attract histone deacetylase activity to the target DNA sites; for that reason, KRAB-ZNF proteins are thought to function primarily as transcriptional repressors [7]. Other members of this family encode different types of effector domains, most commonly SCAN domains (which mediate protein-protein interactions) or BTB domains (also protein-protein interaction domains with potential repressor activity) in addition to the ZNF motifs [8].

In past studies we identified and manually annotated the repertoire of human KZNF genes, and carried out a preliminary comparison with genes in other species [9]. Whereas most KZNF genes are deeply conserved, the KRAB-ZNF subfamily has expanded especially during vertebrate evolution through repeated rounds of segmental duplications, creating many lineage-specific genes [9], [10], [11]. Recently evolved KRAB-ZNF paralogs display remarkable sequence and expression differences, suggesting a drive for functional diversification [12]. This evolutionary diversity, coupled with evidence that KRAB-ZNF genes play a major role in determining gene expression differences between human and chimpanzee brain [13], indicate an important role for the KZNF family, and particularly the KRAB-ZNF subgroup, in primate evolution.

Previous comparisons between members of the KZNF family in humans and other primates have yielded contradictory results. While one study claimed the discovery of a large number of human specific KZNFs [14], another study identified chimpanzee orthologs for all of those putative human-specific KZNF genes [15]. The magnitude of KZNF differences between humans and chimpanzees therefore remains a matter of debate. However, both of these conflicting studies relied on public annotation for identification of KZNF genes. For draft genomes, like those of the non-human primates, these automatically generated, preliminary gene models are often incorrect and comparisons relying upon them can be misleading. Determining the complete content of gene families like the KRAB-ZNFs, most of which are located within regions of tandem segmental duplication, can be especially challenging. Finally, frequent lineage-specific gain and loss of KZNF loci and divergence within the ZNF arrays [9], [12], [16], [17] make ortholog assignment particularly challenging.

To obtain a more accurate picture of primate KZNF diversity, we determined the content of multi-fingered, or “polydactyl” KZNF loci in the reference genomes of chimpanzee, orangutan, and rhesus macaque. This process yielded models for genes including members of the BTB/POZ and other KZNF families in addition to the KRAB-ZNF type, providing a broader look at the evolutionary history of this TF family. We generated high-quality chimpanzee gene models by manual curation and improved the gene models for orangutan and rhesus macaque KRAB-ZNFs by a combination of computational and semi-manual methods. In this paper, we focus on species-specific differences in terms of gene content and sequence divergence that are likely to affect species phenotypes. Our analysis identified a number of species-specific KZNF genes and sequence differences between orthologous primate proteins, demonstrating a clear exception to the overall high degree of similarity in gene content and sequence in primate genomes. Using computational predictions of DNA binding capabilities for the KZNF proteins, we speculate on the impact that human-specific KZNF sequence changes may have had on gene regulatory networks, providing hypotheses that can be tested experimentally in future studies.

## Results

### KZNF clusters are dynamic

The complete set of human KRAB- and SCAN-ZNF loci was annotated previously by our group [9]. Genes of this type have a modular structure in which N-terminal KRAB or SCAN effector domains are encoded intact within a single 5′ exon, and combined with a second, 3′ exon encoding an array of at least 3 tandemly arranged zinc fingers [6]. This modular structure leaves a clear signature of clustered, same-strand motif matches after genome scans with effector and KZNF consensus sequences. Since KRAB-ZNF genes frequently give rise to gene and pseudogene duplicates consisting of only zinc finger arrays [9], [12] we collected and examined all regions in which tandem clusters of 3 or more KZNF motifs were found. This strategy also yielded models for other types of “polydactyl” KZNF genes, including members of the BTB/POZ, zinc finger homeobox and other families, all of which we collected in our public web-based catalog. To facilitate an accurate primate comparison we first updated the human loci reported in our previous study [9] based on new RNA evidence and sequence assembly, including the assignment of new official gene symbols for 10 human genes (Table S1). The current human KZNF gene annotation is available on our updated website (http://znf.igb.illinois.edu).

To enable accurate interspecies comparisons, we used the same approach used previously [9] to identify and manually curate KRAB, SCAN, BTB and other polydactyl KZNF loci in the chimpanzee genome. We also created sets of computationally defined and manually assembled KZNF loci for orangutan and rhesus macaque (see Methods). In this manuscript we will use the term “loci” to describe the total collection of genes and pseudogenes in this family, without reference to coding potential. While a clear distinction between genes and pseudogenes is possible for human loci because of the public availability of mRNA and EST data, such deep transcriptome data is not yet available for the other three primate species in our study. However, manual annotation allows us to distinguish loci with capacity to encode full-length KZNF genes from obvious pseudogenes.

Since the orangutan and rhesus macaque genomes were derived from females we compared the number of loci between species excluding genes located on the Y chromosome. In each species we identified approximately 1000 loci (Table 1). Despite issues related to draft assembly were able to identify orthologs for the majority of (>50%) human genes (Table S2) in each primate species, in addition to several potential species-specific loci using our dataset (see below). The final gene models, including sequences, motif information, and cluster location, for polydactyl KZNF genes from the four primate species are displayed in a browser on our website (http://znf.igb.illinois.edu).

**Table 1:** Total number of KZNF loci, putative protein coding genes, and genes having certain domain compositions identified in four primate species.

Species	All loci	All genes	>1 ED	no ED, ≥3 ZNFs	KRAB-A	SCAN	KRAB-A +≥3 ZNFs	SCAN +≥3 ZNFs	BTB
**Human**	934	609	483	111	404	60	393	54	41
**Chimpanzee**	852	633	493	125	404	61	381	55	42
**Orangutan**	1269	552	368	136	265	42	230	36	70
**Rhesus M.**	851	459	317	110	255	42	245	40	36

Loci on chromosome Y were not included. ED = Effector domain.

**Figure 1:**
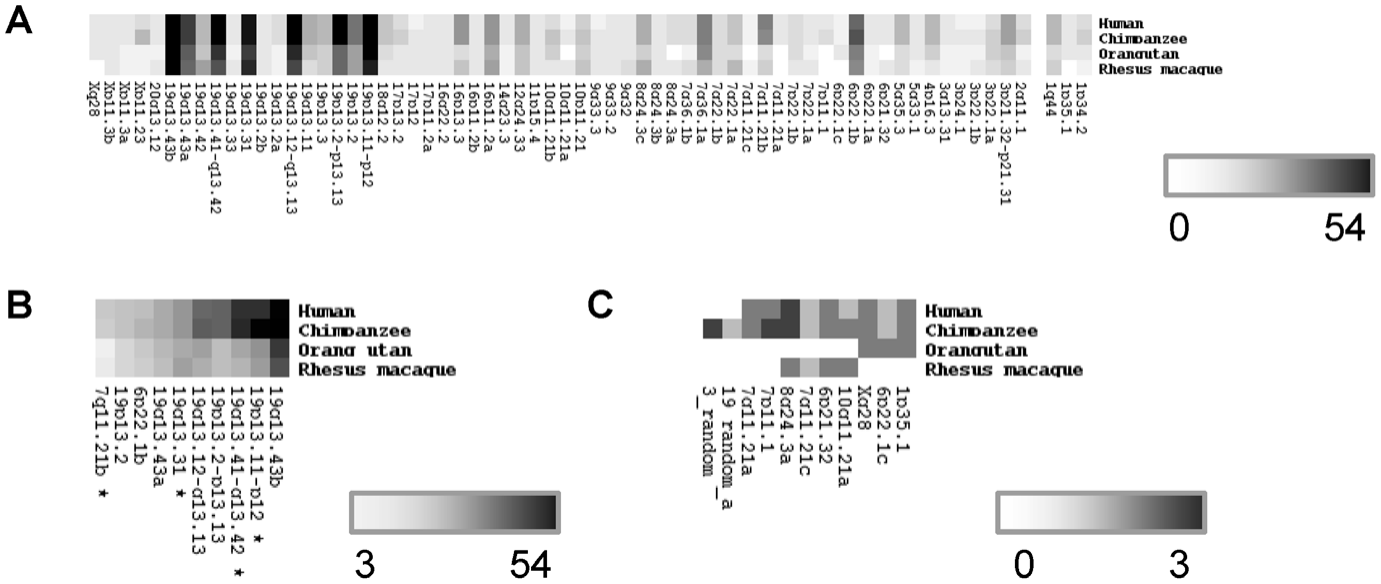
Number of KZNF genes per cluster for the four investigated primates. The legend indicates the number of genes – the darker the fields the more genes are in the cluster. A: All clusters. B: The ten largest clusters. C: Lineage-specific clusters. The asterisk indicates clusters that had orthologs located on random or unknown chromosomes.

By examining open reading frame (ORF) length and motif content (see Methods) we estimate that between 43% (orangutan) and 72% (chimpanzee) KZNF loci detected in each genome are coding genes (Table 1). The majority of loci were found in chromosomal clusters on chromosome 19 in all four species. However, consistent with previous comparisons of human, mouse and dog gene clusters [9], [14], [16], [18] we observed different numbers of loci in almost all clusters in the different primate genomes (Table S3). Not only the total number of loci, but also the number of putative coding KZNFs differed substantially between species in many gene clusters (Figure 1A).

Especially striking differences were observed in the largest human clusters on chromosomes 19, 6 and 7 (Figure 1B). For instance, a cluster located in human chr19p13.11–p12 contains 43 human, 52 chimpanzee, 22 orangutan, and 20 rhesus macaque genes; a second cluster mapping to human chr7q11.21b contains 11 human, 10 chimpanzee, 3 orangutan, and 4 rhesus macaque genes. We also identified whole clusters that are lineage-specific; for instance a cluster on chromosome 7 (7p11.1) contains only loci specific to humans and chimpanzees. Five loci in this cluster (including 2 coding genes) are present in humans and 4 loci (3 genes) are present in chimpanzees (Figure 1C). Furthermore we found two potential human-specific clusters (2q13a and 2q13b), consisting only of KZNF pseudogenes, and four potential chimpanzee specific clusters (19_random_a, 3_random, unknown_a, and unknown_b), containing three and one predicted gene, respectively.

### Frequent gain and loss of KZNF loci

To investigate ortholog relationships in the primate clusters, we devised a strategy that combines three commonly used methods for ortholog identification: Reciprocal Best BLAST hit (RBH), Synteny, and OrthoMCL [19]. We assigned all loci to 1004 orthogroups (“all-inclusive” data set), of which 765 are 1∶1 orthologs between at least two primates and 355 are 1∶1∶1∶1 orthologs between all four primates with “high confidence” (see methods and Table S2). Note that among these 355 loci are 322 human genes, or only 55% of all human KZNF genes.

**Figure 2:**
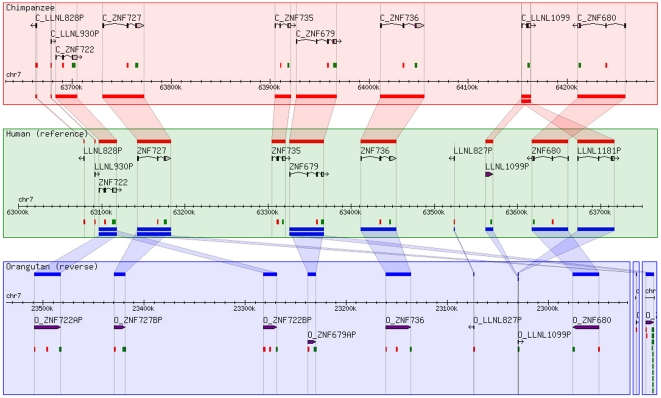
An example of a gene cluster with species-specific loci. Human (cluster chr7q11.21b, region, human chromosome 7∶63000000–63750000) was chosen as the reference for this Gbrowse display. Loci are depicted by rectangles. Orthologous loci are connected by lines. Colors indicate the species comparison: Red for human-chimpanzee and blue for human-orangutan. The human-rhesus macaque comparison is not shown, but can be viewed on our synteny browser (http://znf.igb.illinois.edu/cgi-bin/gbrowse_syn/znf_synteny/). Continuous stretches of chromosomes are shown in a box; orthologs located on other chromosomes (e.g. random chromosomes) are shown in separate boxes. Note the paralogs LLNL1099P and LLNL1181, an example for a human specific duplication, and *ZNF722*, an example for a gene duplicated in orangutan. Further note that for orangutan the reverse is shown, indicating an inversion on the lineage to humans and chimpanzees; the orangutan gene order is likely to be ancestral because it is shared by rhesus macaque (not shown).

The “all-inclusive” dataset allowed us to identify loci that were specifically gained or lost in certain evolutionary lineages (see Methods). Two particular interesting examples are depicted in Figure 2 and Figure S1: the cluster 7q11.21b (Figure 2), which is primate-specific [9], shows considerable differences in locus number between species. The genomic environment of human ZNF705C (Figure S1) is characterized by lineage-specific gene duplicates and smaller scale duplications within genes, as well as an inversion on the human lineage. We created a synteny browser (http://znf.igb.illinois.edu/cgi-bin/gbrowse_syn/znf_synteny/) to display the complete set of these species-specific differences.

In total we identified 74 loci, including 7 predicted coding genes, that are specific to humans and 57 loci (23 genes) specific to chimpanzees (Table 2). To obtain further support for the existence of species-specific loci we compared our data to results of published comparative genomic hybridization (CGH) and whole-genome shotgun sequence detection (WSSD) studies. Eighteen of the 74 loci that we classified as potentially human-specific, including 3 genes (*ZNF658B*, *ZNF84,* and *ZNF492*), were also identified by CGH as loci lost in the chimpanzee compared to human individuals, while only 4 of these loci (1 gene: ZNF286B) showed a higher signal in chimpanzee [20]. Two additional loci (including ZNF286B) were further classified as human-specific by WSSD [21]. Likewise, 19 loci (6 genes) that we identified as potentially chimpanzee-specific gave also a higher signal in CGH in chimpanzee, while only 2 loci (2 genes) gave a lower chimpanzee signal [20], and 2 more of our chimpanzee-specific loci were confirmed by WSSD [21]. These studies give further support for many of our predictions of species-specific loci and genes. Interestingly, 22 loci (5 genes) of the human-specific and 14 loci (3 genes) of the chimpanzee-specific loci were copy-number variant in humans and/or chimpanzees [20], suggesting not only high inter- but also intra-species copy number variability.

**Table 2:** Number of loci and genes gained and lost in a lineage-specific way as well as the number of lineage-specific pseudogenes and proteins with lineage-specific gain or loss of ZNF domains.

Lineage	Gainedloci	Gained genes	Lostloci	Lost genes	Pseudogenized loci	Proteins w/finger gain	Proteins w/finger loss
**H**	74(12.3–16.4)	7(1.2–1.6)	13(2.2–2.9)	0(0)	10(1.7–2.2)	12(2–2.7)	12(2–2.7)
**C**	57(9.5–12.7)	23(3.9–5.1)	46(7.7–10.2)	2(0.3–0.4)	11(1.8–2.4)	4(0.7–0.9)	34(5.7–7.6)
**O**	480(30–40)	145(9.1–12.1)	32(2––2.7)	18(1.2–1.5)	49(3.1–4.1)	17(1.1–1.4)	15(0.9–1.3)
**R**	88(2.7–3.5)	38(1.2–1.5)	n.d(n.d)	n.d(n.d)	40(1.2–1.6)	n.d(n.d)	n.d(n.d)
**HC**	14(0.9–1.2)	4(0.3)	106(6.6–8.8)	40(2.5–3.3)	2(0.1–0.2)	29(1.8–2.4)	69(4.3–5.8)
**HCO**	n.d(n.d.)	n.d.(n.d.)	n.d.(n.d.)	n.d.(n.d.)	1(0.03–0.04)	n.d(n.d)	n.d(n.d)

H = Human, C = Chimpanzee, O = Orangutan, R = Rhesus macaque, HC = Hominines, HCO = Hominids. Note that we cannot distinguish locus gain in HCO from locus loss in R neither place domain changes on the HCO or R lineage. Numbers in parentheses are rates of changes per Million years (My), assuming a divergence time of humans and chimpanzees of 4.5–6 My, of humans and orangutans of 12–16 My, and of humans and rhesus macaques of 25–33 My.

**Figure 3:**
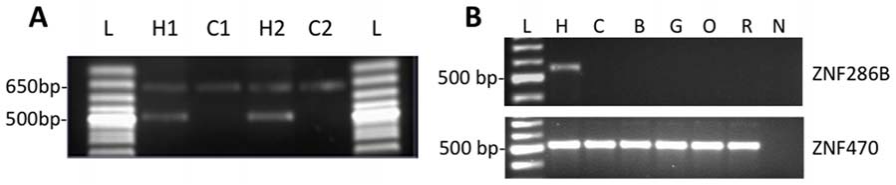
Confirming the human specificity of *ZNF492* and *ZNF286B* genes. **A.** *ZNF492* is predicted to be a human specific duplication of *ZNF98* and can be distinguished from *ZNF98* by several sequence differences, including one mutation that creates a *Bsm*I restriction site in the human-specific gene. We generated PCR products from two independent human (H1, H2) and chimpanzee (C1, C2) genomic DNA samples using primers that would amplify 650 bp regions from both genes and digested the products with *Bsm*I (L = size standard ladder). As predicted, the chimpanzee DNA was not cut by *Bsm*I. By contrast, the human sequence gives rise to three *Bsm*I bands, including the undigested 600 bp *ZNF98* sequence along with 500 bp and 150 bp fragments corresponding to the digested *ZNF492* paralog. The gel shown here was run maximize separation of the 600 and 450 bp bands; the 150 bp fragment is not shown. **B:** *ZNF286B* is predicted to be a human-specific duplicate of *ZNF286A*. We used PCR with the forward primer targeting the first finger that distinguishes the two paralogs to amplify the *286B* gene sequences in genomic DNA from six primates: human (H), Chimpanzee (c), Bonobo (B), Gorilla (G), Orangutan (O), and rhesus macaque (R). A size standard ladder (L) and no-template negative control (N) are also included. A *ZNF286B*-specific PCR product was generated only in human DNA. These same DNA preparations were tested with control PCR primer sets designed against several other genes including *ZNF470,* which is known to be present in all species (lower panel). The production of clear PCR products for this and other shared genes confirmed the quality of the non-human primate DNA.

We also experimentally validated the human-uniqueness of two genes, *ZNF492* and *ZNF286B*. *ZNF492* and its older paralog *ZNF98* can be distinguished by a difference in one DNA-contacting amino acid in the 8^th^ ZNF motif and a mutation of the first cysteine residue in the 13^th^ ZNF motif. One of the sequence differences affects a restriction site for the enzyme *Bsm*I. We used PCR primers common to both *ZNF492* and *ZNF98* to amplify the sequences from 2 human and 2 chimpanzees DNA samples and digested the fragments with *Bsm*I. The restriction digest created only a single, undigested product in chimpanzee representing chimpanzee *ZNF98*. In human samples, however, *Bsm*I digestion yielded three fragments as expected given the presence of both *ZNF98* and *ZNF492* genes (Figure 3A).

In a second example, we looked for the presence of *ZNF286B*, a predicted human-specific copy of a deeply conserved gene, *ZNF286A*. We designed a forward primer within the first ZNF motif that could distinguish between the two paralogs. With the *ZNF286B*-specific primers, PCR amplification yielded a clear band for human, but not for chimpanzee, bonobo, gorilla, orangutan or rhesus macaque (Figure 3B). These data support the inference that *ZNF492* and *ZNF286B* are human-specific genes.

These analyses indicate a surprisingly large number of species-specific loci. In contrast, we found only 14 loci (4 genes) shared by and specific to humans and chimpanzees (hominines, subfamily homininae) (Table 2). This means that during 6–11.5 million years (My) of evolution on the branch from the hominoid ancestor to the homininae ancestor only 0.25–0.35 KZNF genes arose per My, while during the last 4.5–6 My of evolution 1.2–1.6 genes were added per My to the human genome and even 3.9–5.1 genes per My to the chimpanzee genome. Thus the rate of gene gain is about 4.7 and 15 fold higher on the human and chimpanzee lineage, respectively, than on the lineage to their ancestors.

Next we used the “all-inclusive” dataset to identify KZNF loci that might have been lost by deletion specifically in a particular lineage. Thirteen orthogroups contain orthologs of chimpanzee, orangutan, and rhesus macaque, but not human, and could therefore represent loci lost specifically in the human lineage (Table 2). These orthogroups correspond to 13 chimpanzee loci, none of which was classified as a functional gene. Likewise, 46 (2) and 32 (18) loci (genes) are lost from the chimpanzee and orangutan genome, respectively. Among the loci “lost” specifically in chimpanzee are two predicted protein-coding genes, *SCRT2* and *ZNF858*. The number of loci and genes predicted to be lost species-specifically is higher in chimpanzees than humans; the highest rate of gene loss is on the lineage to humans and chimpanzees.

We further identified loci that have degenerated into pseudogenes specifically in one species by analyzing our “high confidence” set of orthologs (Table 2). With 10 and 11 loci being annotated as a pseudogene only in human or chimpanzee, respectively, the rate of pseudogenization is similar in these two species. Two loci pseudogenized on the lineage to humans and chimpanzees and 1 locus is a pseudogene in all analyzed hominoids but not in rhesus macaques (Table 2).

In summary, we observed evolutionary changes in all lineages that considerably changed the content of functional KZNF genes.

### Functional diversification of orthologous KZNF genes

We next analyzed sequence differences between orthologous KZNF proteins. For this purpose we utilized the “high-confidence” dataset, focusing on differences in domain composition, including KRAB, SCAN and finger-encoding regions that are likely to affect protein function. In order to produce high-quality alignments of the ZNF domains we developed a new alignment algorithm (see Methods). In short, this algorithm treats each ZNF motif as a separate unit and attempts to maximize the similarity between pairs of these units in different proteins. This algorithm permits us to identify orthologous ZNF domains and to obtain reliable alignments even in cases where finger duplications or deletions have occurred within the ZNF array, a common path for divergence in this gene family [12], [16], [17].

The most frequent type of species-specific domain change we observed between orthologs is a change in the number of ZNF motifs (Table S4). We observed gain and loss of functional ZNF motifs in each lineage, while the gain or loss of a KRAB, BTB, or SCAN domain in a lineage is very rare. Twenty-four genes are characterized by a human-specific and 38 genes by a chimpanzee-specific alteration in ZNF domain number (Table 2). Interestingly, the rates of ZNF domain gain and loss are highest on the human, chimpanzee, and human-chimpanzee ancestor lineages.

Importantly, we very rarely observed lineage-specific gain or loss of ZNF sequences in pseudogenes. Such mutations were not found in any human and only in three chimpanzee pseudogenes (Table S5), demonstrating that changes in ZNF motif number predominantly affect functional genes (Fisher's exact test, Human: p = 3.83*10^−5^, Chimpanzee: p = 0.0051).

The binding specificity of each ZNF domain is determined by four amino acids directly contacting specific nucleotides [22], and the DNA-contacting residues in KRAB-ZNF gene duplicates frequently show signs of positive selection [16], [18], [23], [24]. For this reason we examined species differences in the DNA-contacting amino acids by comparing ZNF sequences of orthologous KZNF genes. We first tested if certain sites within orthologous ZNF domains evolve under positive selection using the sites models implemented in PAML (see Methods). Only four orthogroups were significant (Likelihood ratio test p<0.05 after correcting for multiple testing): *MYNN*, *GIOT-1*, *ZBTB24*, and *PRDM9*. *PRDM9,* which has been demonstrated previously to be evolving under strong positive selection in primates [25] stands out in that 10/12 significant sites are DNA-contacting and differ between all four primate species. Of the other three genes only *GIOT-1* contains 1 (of 9 total) significant site that is DNA-contacting. The significant DNA-contacting amino acid change in *GIOT-1* represents a rhesus macaque-specific difference.

Finally, we identified all ZNF arrays with lineage-specific sequence differences. About 2% of ZNF motifs have changed in sequence specifically in humans and chimpanzees, about 8% on the lineages to orangutan and homininae, and 16% have changed specifically in rhesus macaques or on the hominoid lineage (Table 3). However, only 8.2% of the changed human ZNF motifs and 6.7% of the changed chimpanzee ZNF motifs demonstrate differences in the sequence of DNA-contacting amino acids (Table S6). Given that 4 out of 28 amino acids (14%) are DNA-contacting, the percentage of changes within these residues is low, indicating avoidance of sequence changes at these positions. In total, only 6 human and 4 chimpanzee proteins harbor species-specific DNA-contacting residues, 4 and 9.5 times fewer proteins, respectively, than all proteins displaying any type of species-specific change in the number of functional ZNF motifs.

**Table 3:** Numbers of ZNF domains with species-specific sequence change.

Lineage	# ZNF domains	# Loci	% ZNF domains	% Loci
**H**	73	56	2.392658	15.51247
**C**	60	50	2.021563	14.32665
**O**	203	115	7.347087	34.7432
**R = HCO**	438	196	15.68768	59.7561
**HC**	242	118	8.554259	34.80826

H = Human, C = Chimpanzee, O = Orangutan, R = Rhesus macaque, HC = Hominines, HCO = Hominids. Note, in this analysis we cannot distinguish between changes on the lineage to rhesus macaque or hominids (R = HCO).

Taken together, these data indicate two main paths through which KRAB-ZNF genes have achieved functional diversification in binding site determination: (1) by changing the number of ZNF domains or (2) by changing the sequence of the DNA-contacting amino acids. Through these paths, 30 human and 42 chimpanzee KZNFs have acquired potential species-specific binding properties. Eleven of these genes have Ka/Ks ratios>1, which suggests accelerated evolution in the genes and positive selection (Table 4). For five of these eleven genes, we could obtain expression information for five human and chimpanzee tissues (30; Figure S2). While ZNF267 and ZNF271 are commonly expressed in all tissues, ZNF470 shows pronounced expression in testis, and ZNF844 is more commonly expressed in testis, kidney, and heart in both species. By contrast, according to these expression data, ZNF780B is excluded from chimpanzee brain and heart, while it is clearly expressed in these tissues in humans.

**Table 4:** KZNF genes with human or chimpanzee specific changes in ZNF domains and Ka/Ks>1.

Gene	Ka/Ks	DNA sequence identity ZNF domains	Species-specific change
**ZNF267**	99	99.83	Human-specific DNA-contacting amino acid
**ZNF780B**	99	99.50	Human-specific DNA-contacting amino acid, Chimpanzee-specific ZNF domain loss
**ZNF749**	99	97.62	Chimpanzee-specific ZNF domain loss
**ZNF895**	99	99.40	Chimpanzee-specific ZNF domain loss
**ZNF389**	99	99.60	Human-specific ZNF domain loss
**ZNF470**	99	99.70	Human-specific ZNF domain gain, Chimpanzee-specific ZNF domain loss
**ZNF534**	2.31	99.36	Human-specific ZNF domain gain
**ZNF844**	1.59	98.81	Human-specific ZNF domain loss
**ZNF860**	1.37	96.65	Chimpanzee-specific ZNF domain loss
**ZNF271**	1.04	99.29	Human-specific ZNF domain loss
**ZNF788**	1.02	99.32	Human-specific ZNF domain loss, Chimpanzee-specific ZNF domain gain

### Prediction of target genes

**Figure 4:**
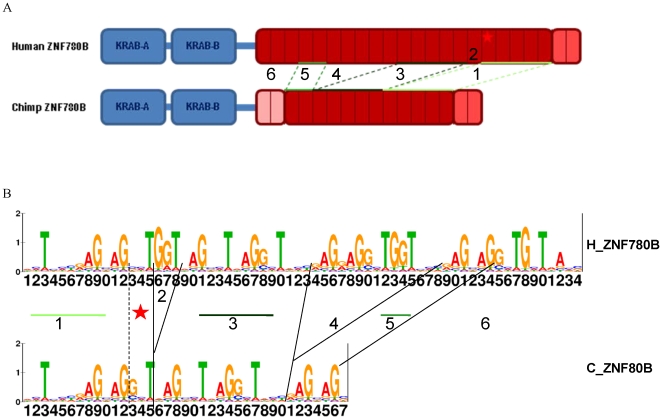
Sequence and predicted binding motif differences between human and chimpanzee ZNF780B. **A.** Both proteins contain a KRAB-A, a KRAB-B, and a polydactyl ZNF domain. The last two ZNF motifs are not functional in both species due to mutations of the Histidine residues preventing the correct folding of the binding domain. The first two ZNF motifs of the chimpanzee gene are not included in the protein. Homologous ZNF motifs are indicated by green lines. The first three, the 6^th^ to 10^th^, as well as the 16^th^ ZNF motif are deleted in chimpanzee ZNF780B. The human ZNF780B protein is characterized by a human-specific amino acid at position 6 in the 17^th^ ZNF motif, indicated by a star. **B:** Binding motifs for human and chimpanzee ZNF780B as predicted by the tool developed by Kaplan and colleagues (28). Corresponding stretches of the binding motif and ZNF domains are indicated by numbers, the star, and green lines, respectively.

We previously predicted that a few changes in the ZNF array between *ZNF417* and its paralog *ZNF587* (one ZNF gain and two differences in DNA-contacting amino acids) should lead to dramatic functional differences between the two closely related genes [12]. We used a similar approach to investigate potential functional consequences for a not yet functionally characterized KZNF gene displaying species-specific structural changes: *ZNF780B*. The ZNF780B protein contains a human-specific change in a DNA-contacting amino acid in its 17^th^ (out of 21) functional ZNF motif compared to other primates. In addition the orthologous chimpanzee gene has specifically lost 9 ZNF domains, an array of five internal domains, a single internal domain, and three terminal domains, through deletions in the ZNF array (Figure 4A). The human-specific change appears to be fixed as it is not reported to be a SNP in dBSNP (build 131).

What could be the impact of these evolutionary changes? To provide a possible answer to this question, we used a tool for predicting ZNF binding sites based on the four DNA-contacting amino acids of individual ZNF domains [26] (Figure 4B). We coupled these predictions with genome-wide scans for these predicted binding sites, expression and network analysis to infer species differences in potential targets between the orthologous TFs (see Methods). The human and chimpanzee versions of the predicted ZNF780B DNA binding motif are considerably different (Figure 4B). The most consequential changes in the predicted binding motif are due to the human-specific DNA-contacting amino acid that removes the high preference for a G at position 13 (indicated by the star in Figure 4B) and due to the chimpanzee-specific ZNF-domain deletions, which remove three segments of the binding sequence (indicated by 2, 4, and 6 in Figure 4B).

Importantly, segments 2 and 4 correspond to three and 15 nucleotides, respectively, that are located at internal positions of the binding site. These deletions would therefore be predicted to put downstream fingers in the chimpanzee protein out of register with the human binding motif. While the human and chimpanzee proteins could bind common targets using short subsets of conserved fingers, the preferential binding sites for these two proteins, and their relative stability at particular binding sites, are nonetheless predicted to be considerably different.

We found the predicted preferred human ZNF780B motif near five genes in the human genome: three genes with no official Gene symbol and *DAB1* and *OLIG3*, both important for neuronal function and development. By contrast, the chimpanzee ZNF780B DNA binding motif occurs much more often (217 times) in the genome due to its shorter length. An analysis of enrichment of Gene Ontology terms [27] revealed that many of the genes located near the chimpanzee ZNF780B motif in the chimpanzee genome are involved in the immune system or are involved in neurotransmitter function. These predicted functions are particularly interesting, given the differential expression of *ZNF780B* in human and chimpanzee brain (see above).

Five genes were in the vicinity of the predicted chimpanzee ZNF780B motif in both the chimpanzee and human genomes: *NFIC* (involved in tooth and central nervous system development and aging [28], [29]), *PDGFC*, *C6orf195*, *SLC6A1* (a GABA transporter), and *SPRY1* (involved in cranio-facial, cardiac, neural crest, adipocyte, limb and muscle development, and in neurogenesis [30], [31], [32]). However, due to the sequence divergence of the human and chimpanzee ZNF780B binding domain, it is possible, that these genes are not regulated by human ZNF780B.

To put these predicted targets in a functional context we gathered more information about their interaction partners (see Methods). A network deduced for the predicted human and chimpanzee ZNF780B targets is shown in Figure S3. In human, *DAB1* and *OLIG3* are connected by *TCF12*, a protein important for neurogenesis [33]. Interestingly, only *TCF12* is significantly correlated in expression with *ZNF780B* across five human tissues (Spearman's rank correlation, p<0.05 after Bonferroni correction). Likewise for chimpanzee *ZNF780B*, we identified 3 correlated genes (without correction for multiple testing), *GRB2, NFIC*, and *SLC6A1*, giving further support that *NFIC* and *SLC6A1* are indeed regulated by *ZNF780B* in chimpanzees.

## Discussion

We have significantly improved the information on content and the quality of gene models for KZNF genes in primate species, and have used this information to identify species-specific gain, loss and sequence divergence in KZNF genes and pseudogenes. We cannot rule out the possibility that some loci that we call “lost” are still “hidden” in the unfinished genomes. The human-specific differences we report here are of especially high confidence, both because of the finished status of the human genome and the low likelihood of finding the same sequence error independently in three other primate genomes. But also across all analyzed primates the differences are pronounced enough to demonstrate clearly that the KZNF gene clusters have undergone pronounced changes in recent primate history. Most remarkable are the speed at which new KZNF genes are added to individual species lineages and the high levels of variability in ZNF domain composition between species. This high degree of differences in gene content and duplication/deletion of binding domains is in stark contrast to the high sequence similarity in the alignable parts of the primate genomes (e.g. ∼99% between humans and chimpanzees). Further, we show that the most common path for acquiring potential species-specific functionality is through changes in the number of DNA-binding ZNF motifs. Finally, we performed analyses that allow predictions to be made regarding functional consequences of changes in one protein, *ZNF780B*.

The rate of species-specific KZNF gene gain is higher than the rate of species-specific KZNF gene loss in hominids, although considerable numbers of genes have been lost by species-specific pseudogenization. Interestingly, the rate of gene gain increased during very recent evolution and was highest on the individual species lineages. We observed the highest rate of gene loss and the highest rate of changes in ZNF domain number between orthologs, on the lineages to humans, chimpanzees, and the human-chimpanzee ancestor. These data indicate that the KZNF gene repertoire has been re-shaped dramatically during the last ∼14 Myr.

For PRDM9, a KRAB-ZNF gene predicted to be involved in recombination hotspot evolution and speciation [34], [35], [36], it was recently shown that especially the DNA-contacting amino acids of its ZNF domains differ between primate orthologs and show signs of positive selection [25]. However, in general for the KZNF family, we found more species differences in the number of ZNF motifs than in the sequence of the DNA-contacting amino acids in the orthologous protein-coding genes. The high number of species differences in the DNA-contacting amino acids therefore makes the *PRDM9* protein a striking exception. In this respect, the divergence path for KZNF orthologs differs from that of recently duplicated primate KZNF paralogs, which displayed evidence of positive selection in the DNA-contacting amino acids [12].

However, differences in the number of ZNF motifs could potentially have even more pronounced functional impact than amino acid changes, because in theory each ZNF motif can bind to three adjacent nucleotides [22]. ZNF motif duplications might therefore create a different or possibly a more energetically stable binding site. On the other hand, ZNF deletions could reduce the stability or specificity of the protein permitting more promiscuous DNA binding. Finally, duplications or deletions of internal ZNF motifs could put neighboring finger motifs “out of register” with their target sites, and could therefore completely alter the binding site of a KZNF. Our finding that changes in ZNF number occur significantly more often in genes than in pseudogenes points to positive selection for functional divergence between primate KZNF genes by changes in ZNF domain number.

Transcription factors typically cooperate in networks and evolutionary changes in TF gene number and sequence can therefore be predicted to be highly consequential for gene regulatory network (GRN) connectivity and structure. In theory, the new duplicate will initially regulate the same target genes as the parental copy. However, later diversification can permit the novel TF protein to acquire unique binding sites and target genes [2]. Changes in the ZNF motif number and/or sequence can be predicted to influence target choice, providing a path to GRN rewiring. Interestingly, several of the KZNFs displaying human or chimpanzee-specific changes showed signs of positive selection, indicating that the sequence changes have been functionally consequential and have provided an advantage to either primate species.

Since the functions and targets of most KZNFs are unknown, we can only speculate about the effects of these sequence changes on the GRN. However, taking advantage of the predicted relationships between zinc-finger motif sequence and DNA binding sites, known protein interactions, and information on co-expression partners, we present here a set of testable hypotheses about the regulatory effects that have accompanied human-chimpanzee sequence changes in one KZNF gene, *ZNF780B*. Although functional studies will be needed to confirm these hypotheses, it is intriguing that many predicted consequences affect the development or regulation of the nervous system, muscles, limbs or teeth, features that indeed differ between humans and other primates.

Our study demonstrates the power of manual annotation of gene models. Automatically derived gene models can give profoundly misleading results for genes with high sequence divergence or copy number variation between species. Our data point clearly to sets of lineage-specific TF genes, that together are likely to have played a role in the evolution of primate morphological and physiological traits. The ready availability of genome-wide methods such as chromatin-immunoprecipation (ChIP) and siRNA knockdown methods open the doors to direct testing of these hypotheses in future studies.

## Materials and Methods

### Identification and annotation of primate KZNFs

The chimpanzee genome (PanTro2) including “random chromosomes” was downloaded from UCSC. The genome was translated into all six frames and scanned for the following functional domains using HMMER: KRAB-A, KRAB-B, KRAB-b, KRAB-BL, KRAB-C, ZNF, SCAN, BTB. HMMER matrices were taken from [9]. Loci were then annotated using Apollo [37] if at least one KRAB motif had an E-score of <10^−6^ or if at least three ZNF domains were in close proximity (<100 bp genomic distance). Loci with SCAN or BTB domains were only annotated if at least three ZNF domains were in close proximity to them. We required that the order of the domains in these models be as described previously for KRAB-ZNFs [6] with an exon coding for the ZNF array located 3′ of all other domain coding exons, and the KRAB-A encoding exon is located 5′ of other types of KRAB domain exons. Open reading frames (ORF) and exon-intron boundaries were set manually. 5′ and 3′ UTRs were inferred from human 1∶1 orthologs. All chimpanzee models including coordinates, protein sequence, coding sequence, and UTR sequences can be downloaded from our website, http://znf.igb.illinois.edu.

The orangutan (PonAbe2) and rhesus macaque (RheMac2) genomes were downloaded from UCSC and scanned for motifs as described above. Domains were assembled to crude loci as described in [9]. We only assembled loci if they fulfilled the same conditions for E-score, domain distances, domain order and ZNF domain numbers as described for chimpanzee loci. However, ORFs and splice sites were not determined; rather the domain coding exons were extended 5′ and 3′ until the next stop codon. These gene models are displayed at http://znf.igb.illinois.edu.

We estimated the number of KZNF genes by counting all loci that have an open reading frame (ORF) of at least 200 amino acids (aa) which would be long enough, for instance, to encode at least one KRAB and three ZNF domains. For the human genome this estimate is close to the known number of “polydactyl” KZNF genes in our catalog (http://znf.igb.illinois.edu/hg18) and overestimates by only 20 genes (3%).

Throughout this manuscript we use the official gene symbols for genes and pseudogenes assigned by the Human Genome Organization (HUGO) for the human loci. The naming scheme for loci in the other three primates is explained in Text S1.

### Individual genome and synteny browser

Original gene model information were obtained from the UCSC releases for human (hg18), chimpanzee (panTro2), orangutan (ponAbe2), and rhesus macaque (rheMac2). All UCSC gene models (including those from RefSeq and Ensembl) as well as our local KZNF models were converted to GFF3 format for loading into species-specific Bio::DB::SeqFeature::Store databases [38], [39]. These databases serve as backend storage for visualization of genome features by the Generic Genome browser (GBrowse) [38].

A sparse synteny database was created by pairwise joining of gene region information for KZNF orthogroups and was used as the joining database for visualizing comparisons between all four genomes using GBrowse_syn [40].

### Ortholog assignment

Orthologs for all loci were identified based on a combined approach of reciprocal best BLAST hit (RBH), synteny, and OrthoMCL [19] and assigned to orthogroups (consisting of orthologous and in some cases paralogous loci as described below). For all pairwise species combinations RBHs of DNA sequences were determined using a custom Perl script. To identify loci at homologous genome locations we used the UCSC Liftover (LO) tool. All three methods identified fewer 1∶1 (:1∶1) orthologs with increasing evolutionary distance (Table S7). While RBH and OrthoMCL largely agreed, a considerable number of these putative orthologs were not located at synthenic genome positions. Consistent results were obtained from all three methods for 657 human-chimpanzee 1∶1 orthologs and for 234 1∶1∶1∶1 orthologs for which all pairwise combinations of all 4 primate species were consistent. These 234 orthogroups therefore contain the most conserved primate KZNF loci.

Because this method is likely to be an underestimate of the number of orthologs we manually added orthogroups in a step-wise fashion. First, orthologs were grouped for which all three methods gave consistent results for a subset of the species. This formed 765 orthogroups, among them 355 groups that have orthologs in all four species. We call this set of orthologs the “high-confidence” set. Next, we added orthologs if they were RBH or at 1∶1 LO coordinates between two species and were grouped by OrthoMCL in the same group or not grouped in any group at all. This allowed inclusion of more diverged loci and of loci in re-arranged or misassembled genomic regions. This step increased the number of orthogroups to 838. Further orthologs were added if they were the best BLAST hit in one species or at LO coordinates of another species and grouped by OrthoMCL in the same group or not grouped in any group at all; increasing the number of orthogroups to 854. Finally, loci were added that were 1∶1 within-species best BLAST hits if they were grouped by OrthoMCL in the same group or not grouped in any group at all. In this last step we assigned each locus to an orthogroup, totaling the number of orthogroups to 1004. This “all-inclusive” set contains orthogroups that can have species-specific paralogs, i.e. 1:many and many:many relationships. For our analyses we used the “high-confidence” or “all-inclusive” set depending on the question we were investigating.

### Lineage-specific loci gain and loss

Orthologous clusters were identified based on all high-confidence 1∶1 orthologs between pairs of species (see above). This determined which clusters contain orthologous genes and allowed to add loci from “random” and “unknown” chromosomes to the comparison of loci numbers between orthologous clusters. Due to the lack of transcript information from non-human primates, we defined loci as pseudogenes if their sequence is shorter than 150 amino acids or if they had a stop codon within the first 600 nucleotides, which essentially means loci representing ORFs smaller than 200 amino acids.

We assigned lineage-specific gain of loci the following way: first we identified orthogroups of the “all-inclusive” set that contained unequal numbers of loci in the different species. Additional loci are likely to represent species-specific paralogs, because they show more sequence similarity to the locus of the same species than to the orthologous loci in the orthogroups. We then excluded the paralogs that had 1∶1 orthologs in the “high confidence” set to determine which loci were lineage-specifically gained. For example, in an orthogroup having 1 human, 1 chimpanzee, 2 orangutan, and 1 rhesus macaque loci it is likely that there was a locus gain on the orangutan lineage. To assign lineage-specific loss of loci we identified orthogroups of the “all-inclusive” set that missed orthologs of a particular lineage. For example, in an orthogroup having 2 human, 2 chimpanzee, 1 orangutan, and 2 rhesus macaque loci it is likely that there was a locus loss on the orangutan lineage. We cannot distinguish between rhesus-specific locus gain and locus loss on the hominoid lineage. To identify orthogroups with lineage-specifically pseudogenized loci, we used the “high confidence” dataset.

To obtain further support for human- and chimpanzee-specific loci, we intersected the genomic coordinates of the KZNF loci with the genomic coordinates of regions identified to have copy number changes between humans and chimpanzees or to be copy number variant in either species as determined by CGH [20] and WSSD [21]. We are reporting the loci that overlapped with the coordinates of either study.

### Experimental testing for human-specific genes

We designed the following primers to amplify ZNF492/739: Forward: 5′ TTCATGCTGGAGAGAAACCT 3′;

Reverse: 5′ ATGTCTGTTAAGAATAGAGGAGTTGT 3′. We performed 25 µl PCR reactions with 0.125 polymerase using 72°C annealing temperature and 2 min extension time. Resulting PCR products were purified using the QIAquick PCR Purification Kit and digested with restriction enzyme Bsm1 in NEBuffer 4 for 60 minutes at 65°C. Digestion products were run on a 2% agarose gel together with O'RangeRuler 50 bp DNA Ladder.

For ZNF286B we designed specific primers matching only human ZNF286B but not ZNF286A: Forward: 5′GCCATTCAGTGCATATTCAACACCAG 3′;

Reverse: 5′TACACTCATAGGGTTTCTCTCCAGTGTGA 3′. We performed 20 µl PCR reactions with 5PRIME Perfect*Taq*™ DNA polymerase using 69°C annealing temperature and 1 minute extension time. PCR products were run on 1.8% agarose gels together with the Axygen 100 bp Ladder. We should note that we successfully performed PCR reactions for other target regions using the same DNA preparations, so that the absence of a PCR product for ZNF286B in the non-human primates cannot be explained by bad DNA quality.

### Motif-Aligner

We developed a custom program (MotifAligner) to align the protein sequences of canonical ZNF domains. MotifAligner performs a global alignment with affine gaps. In contrast to commonly used alignment programs, MotifAligner operates on whole motifs as opposed to single nucleotides or amino acids. Specifically, two motif sequences T* = t_i_* and U = *u_j_* are aligned by first computing all pairwise motif similarity scores 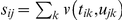, with *v* returning values from an amino acid substitution matrix and k being the motif length. The similarity scores are then used by an implementation of the Needleman-Wunsch algorithm [41] to align the motif sequences. Note that the outcome of the alignment is determined solely by the protein sequence of the motifs; protein sequence outside the motifs is ignored. MotifAligner has been used with a BLOSUM85 amino acid substitution matrix, a gap opening penalty of motif-length^−1^, and a gap extension penalty of motif-length^−0.9^, where motif-length equals 84, which is the number of nucleotides of a ZNF motif.

### Assignment of lineage-specific domain differences

We used the set of “high-confidence” orthologs to analyze differences in domain composition and ZNF sequence. Included were loci with orthologs present in at least three species. This allowed us to identify in which lineage (human, chimpanzee, orangutan, or homininae (human and chimpanzee)) the sequence difference occurred. We cannot distinguish domain gains (losses) in the rhesus macaque from losses (gains) in the hominidae (great apes) lineage.

We used the sites models, LRT 1vs2 and 7vs8, implemented in PAML [42] to test in the orthologous finger regions of the 295 high-confidence orthologous genes with ZNF domains if certain amino acids show signs of accelerated evolution suggestive of positive selection. P-values were corrected for multiple testing using the Bonferroni method. We report sites with a posterior probability of p>0.95 as significant.

### Target prediction

We extracted the four DNA-contacting amino acids of functional ZNF domains of human and chimpanzee ZNF780B and used the tool developed by Kaplan and colleagues [26] to predict binding sites for KZNF genes. The human (HG18) and chimpanzee (PanTro2) genomes were scanned for the predicted binding motifs:

H_ZNF780B: AGNNTGGTNAGNNTNAGGNTNNNNAG;

C_ZNF780B: AGGNTNAGNNTNAGGNTNNNNAG;

The R package ChIPpeakAnno was utilized to assign binding sites to the closest Ensembl gene. The Cytoscape plugin BisoGenet [43] was used to predict interaction partners of the proteins with human and chimpanzee ZNF780B binding motif. Further, Affymetrix microarray data of five human and chimpanzee tissues pre-processed as in [23] were used to identify co-expressed genes of human and chimpanzee ZNF780B.

## Supporting Information

Figure S1 **A snapshot of human chromosome 8:11870862-12350861 compared to the related chimapanzee region in the synteny browser**. This example highlights a region with whole gene duplication in addition to smaller duplications affecting domain architecture. Human was chosen as the reference for this display. Loci are depicted by rectangles. Orthologous loci are connected by lines. Colors indicate the species comparison: Red for human-chimpanzee, blue for human-orangutan. Human *ZNF705C* and *ZNF705D* are closely related paralogs that most likely originated from a human-specific gene duplication. Both consist of one KRAB-A, one KRAB-B and 7 ZNF domains (of which 4 are present in the protein). The tree below the map shows all related orthologous loci in this subfamily. There is only one genomic region orthologous to the two human genes. In this region, a small duplication occurred that involved the KRAB-B domain. This genomic region can now produce three different transcripts, that include all KRAB domains (ABB, C_ZNF705CB), both KRAB-B but only part of the KRAB-A domain (BB, C_ZNF705CA), or only one of the KRAB-B domains (AB, C_ZNF705CC). A further small duplication in the chimpanzee genome added one ZNF domain to the *ZNF705C* locus, so that 5 ZNF domains are present in the protein. The duplications in the chimpanzee genome cause the chimpanzee *ZNF705C* to be most distantly related to the human *ZNF705C* paralogs than any of the other orthologs. A number of orangutan specific duplications resulted in 5 copies of *ZNF705C*, which are found in 5 different genomic locations, all un-assembled. The most complete copy is O_ZNF705CE, which encodes KRAB-A, B, and 7 ZNF domains like human ZNF705C. All other copies miss either KRAB- or ZNF domains. It is unclear at present if the extra orangutan copies represent pseudogenes or real genes that are just not properly assembled yet. There is one *ZNF705C* ortholog in the orangutan genome that has lost both KRAB domains but has 7 ZNF domains like human *ZNF705C*. Note that the chimpanzee, orangutan, and rhesus macaque genomic regions are shown in reverse, indicating a human-specific inversion.
(TIF)

Figure S2 **Expression of KZNF genes with human or chimpanzee specific ZNF domain changes and Ka/Ks>1.** Out of eleven such genes we could obtain expression information from five human and chimpanzee tissues for five genes. The darker the field, the higher the percentage of individuals (ranging from 0 to 100%) expressing the gene in a given tissue.
(TIF)

Figure S3 **Interaction network of genes with ZNF780B binding motif (A: human, B: chimpanzee).** Blue links represent known protein-protein interactions; red lines represent known gene regulatory interactions.
(TIF)

Table S1 **Ten genes that were not assigned official gene symbols by the Human Genome Organization (HUGO) before publication of this manuscript.**
(XLSX)

Table S2 **Pair wise number of high-confidence and all-inclusive orthologous loci and number of orthologous loci in hominids and all four primates.**
(XLSX)

Table S3 **Number of KZNF loci in orthologous clusters of human, chimpanzee, orangutan, and rhesus macaque.**
(XLSX)

Table S4 **For the different types of functional domains in KZNFs the number of loci that gained or lost such domains in a lineage-specific way are given.**
(XLSX)

Table S5 **A list of loci with lineage-specific ZNF domain composition.**
(XLSX)

Table S6 **ZNF domains with human or chimpanzee specific DNA-contacting amino acids.**
(XLSX)

Table S7 **Number of orthologs based on best reciprocal blast hit (RBH), synteny, and/or OrthoMCL.**
(XLSX)

Text S1 **Gene and Pseudogene Nomenclature**
(DOCX)
