# Supplementary figures and images for: Gain, Loss and Divergence in Primate Zinc-Finger Genes: A Rich Resource for Evolution of Gene Regulatory Differences between Species
Source: PLoS One. 2011 Jun 29;6(6):e21553. doi: 10.1371/journal.pone.0021553 (PMC3126818; doi:10.1371/journal.pone.0021553)

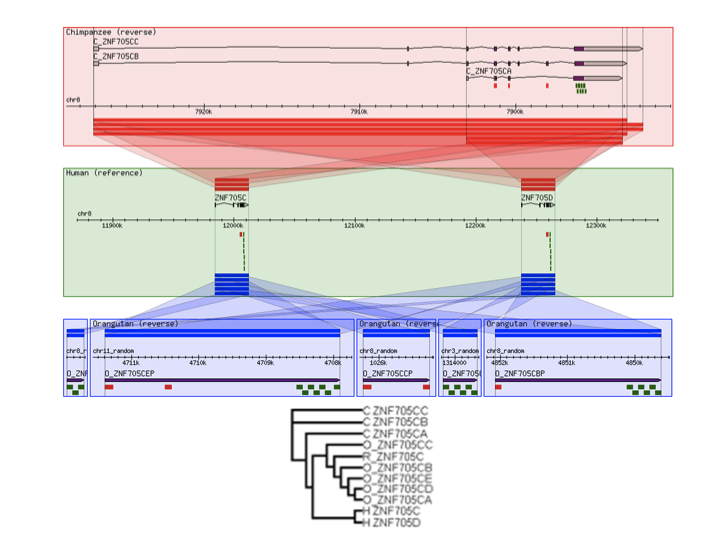

Supplement: Figure S1 — A snapshot of human chromosome 8:11870862-12350861 compared to the related chimapanzee region in the synteny browser. This example highlights a region with whole gene duplication in addition to smaller duplications affecting domain architecture. Human was chosen as the reference for this display. Loci are depicted by rectangles. Orthologous loci are connected by lines. Colors indicate the species comparison: Red for human-chimpanzee, blue for human-orangutan. Human ZNF705C and ZNF705D are closely related paralogs that most likely originated from a human-specific gene duplication. Both consist of one KRAB-A, one KRAB-B and 7 ZNF domains (of which 4 are present in the protein). The tree below the map shows all related orthologous loci in this subfamily. There is only one genomic region orthologous to the two human genes. In this region, a small duplication occurred that involved the KRAB-B domain. This genomic region can now produce three different transcripts, that include all KRAB domains (ABB, C_ZNF705CB), both KRAB-B but only part of the KRAB-A domain (BB, C_ZNF705CA), or only one of the KRAB-B domains (AB, C_ZNF705CC). A further small duplication in the chimpanzee genome added one ZNF domain to the ZNF705C locus, so that 5 ZNF domains are present in the protein. The duplications in the chimpanzee genome cause the chimpanzee ZNF705C to be most distantly related to the human ZNF705C paralogs than any of the other orthologs. A number of orangutan specific duplications resulted in 5 copies of ZNF705C, which are found in 5 different genomic locations, all un-assembled. The most complete copy is O_ZNF705CE, which encodes KRAB-A, B, and 7 ZNF domains like human ZNF705C. All other copies miss either KRAB- or ZNF domains. It is unclear at present if the extra orangutan copies represent pseudogenes or real genes that are just not properly assembled yet. There is one ZNF705C ortholog in the orangutan genome that has lost both KRAB domains but has 7 ZNF domains like human ZN [file pone.0021553.s001.tif]

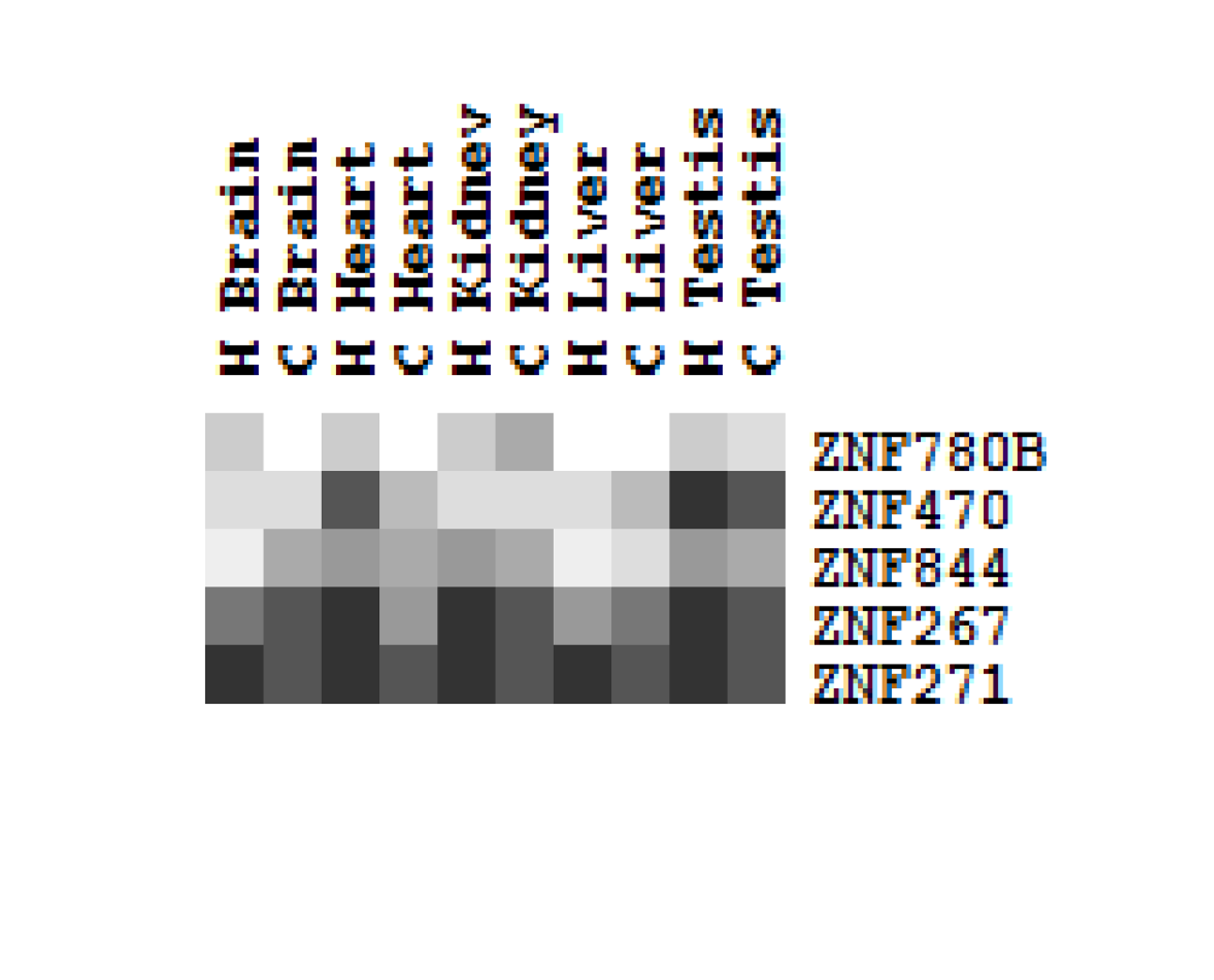

Supplement: Figure S2 — Expression of KZNF genes with human or chimpanzee specific ZNF domain changes and Ka/Ks>1. Out of eleven such genes we could obtain expression information from five human and chimpanzee tissues for five genes. The darker the field, the higher the percentage of individuals (ranging from 0 to 100%) expressing the gene in a given tissue. (TIF) [file pone.0021553.s002.tif]

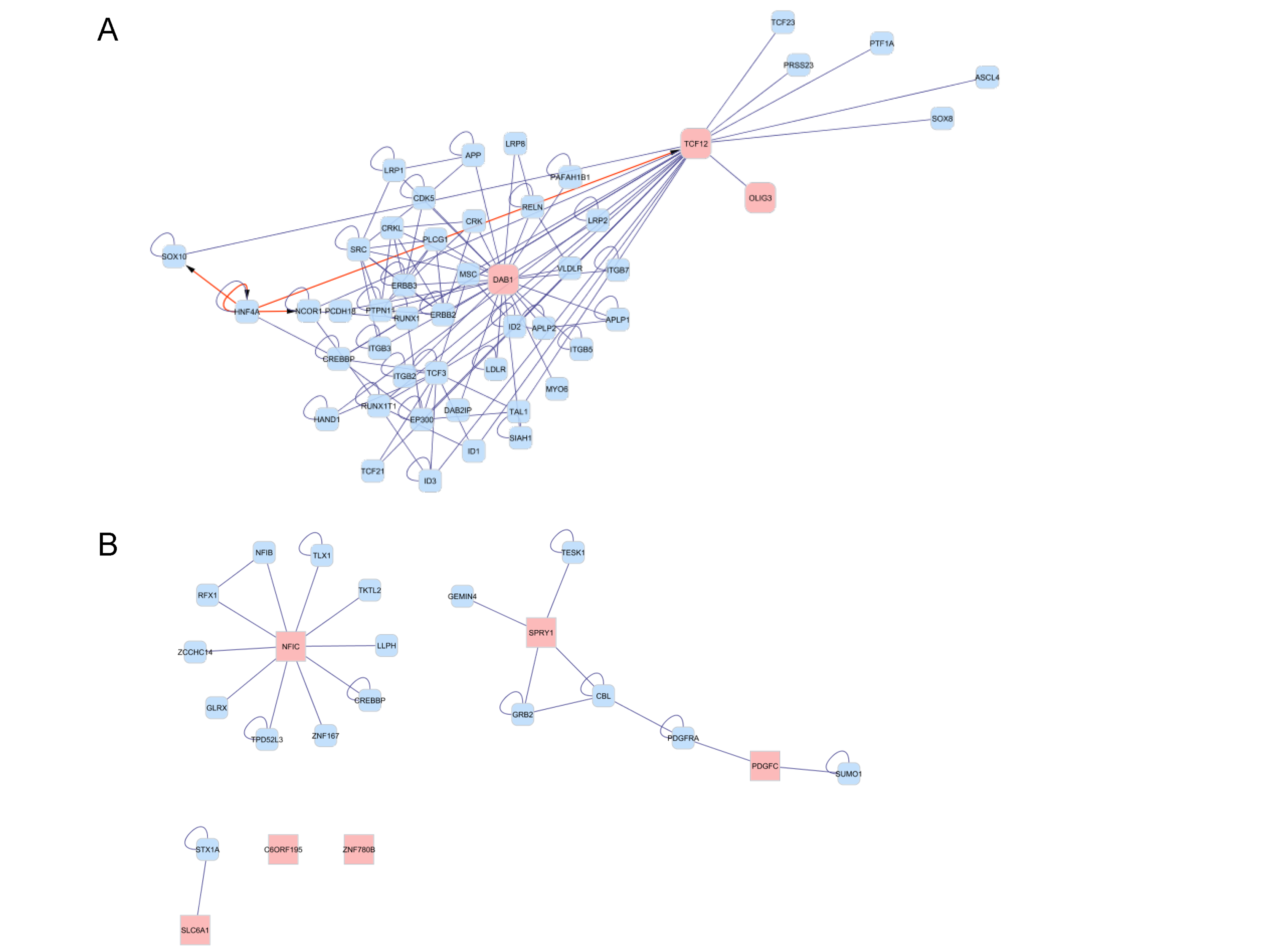

Supplement: Figure S3 — Interaction network of genes with ZNF780B binding motif (A: human, B: chimpanzee). Blue links represent known protein-protein interactions; red lines represent known gene regulatory interactions. (TIF) [file pone.0021553.s003.tif]
