# Supplementary material for: Gain, Loss and Divergence in Primate Zinc-Finger Genes: A Rich Resource for Evolution of Gene Regulatory Differences between Species
Source: PLoS One. 2011 Jun 29;6(6):e21553. doi: 10.1371/journal.pone.0021553 (PMC3126818; doi:10.1371/journal.pone.0021553)
Supplement: Text S1 — Gene and Pseudogene Nomenclature (DOCX) [file pone.0021553.s011.docx]

# Text S1

Human KZNF gene names are the official gene symbols given by the Human Genome Organization (HUGO). For ten genes with no official human gene symbol yet, HUGO assigned new gene symbols (personal communication with Matt Wright; Table S1). Some pseudogenes have had an official name assigned; all other pseudogenes start with the prefix LLNL (from our previous KZNF catalog; Huntley et al., 2006). All pseudogenes have the suffix “P”. We assigned to all 1:1 orthologs the human (pseudo)gene name and added the prefix “C_”, “O_”, or “R_” to indicate the species. If more than one ortholog exists, a suffix “A”, “B” … was added. For loci with no human ortholog, we first assigned consecutive IDs starting with C_LLNL1300 to chimpanzee loci and named the orthologs accordingly by adding prefixes to indicate the species and suffixes to indicate 1:many orthologs and pseudogenes. Remaining orangutan loci were named by consecutive IDs starting with O_LLNL1400, and rhesus orthologs were named accordingly. The remaining rhesus macaque loci were named starting with R_LLNL1500.
